# PROVIDER PERCEPTIONS OF FACTORS IMPACTING COUPLES’ EMOTIONAL DISTRESS AND ADJUSTMENT TO DEMENTIA DIAGNOSES

**DOI:** 10.1093/geroni/igad104.0428

**Published:** 2023-12-21

**Authors:** Sarah Bannon, Julie Brewer, Nina Ahmad, Talea Cornelius, Robert Parker, Bradford Dickerson, Christine Ritchie, Ana-Maria Vranceanu

**Affiliations:** Mount Sinai Hospital, New York City, New York, United States; Massachusetts General Hospital, Boston, Massachusetts, United States; Massachusetts General Hospital, Boston, Massachusetts, United States; Columbia University Irving Medical Center, New York City, New York, United States; Massachusetts General Hospital, Boston, Massachusetts, United States; Massachusetts General Hospital, Boston, Massachusetts, United States; Massachusetts General Hospital, Boston, Massachusetts, United States; Massachusetts General Hospital, Boston, Massachusetts, United States

## Abstract

Alzheimer’s disease and related dementias (ADRD) are increasingly common conditions that disrupt the lives of persons living with dementia and their spousal care-partners. At the time of ADRD diagnoses, many couples experience challenges that produce emotional distress and relationship strain. At present there are no interventions to address these challenges early after diagnosis to promote positive dyadic adjustment. As the first phase of a larger study, we conducted a qualitative study to elicit and systematically summarize the perspectives of ADRD medical stakeholders (e.g., neurologists, geriatricians, social workers, neuropsychologists, care coordinators) surrounding common factors impacting distress following ADRD diagnoses. Participants (n=15) were recruited from two U.S. academic medical centers and completed a 60-minute focus group or individual interview that was transcribed, deidentified, and analyzed using a rapid data analysis approach to thematic analysis. We identified findings within 3 overarching themes: reactions to diagnosis, changes in relationship roles/dynamics, and insufficient resources. Participants described couples’ distress surrounding one partner’s denial or lack of insight into the diagnosis and symptoms, experiences of loss and hopelessness, and uncertainty surrounding illness trajectory. They noticed changes in couples’ historic roles, social determinants of health, and divergent understandings and reactions to diagnosis as contributors to couples’ distress. Finally, providers observed unmet needs surrounding embedded counseling, care-planning, and financial support for current and future care needs. Most providers felt insufficiently prepared to help couples experiencing distress. Our findings underscore the importance of improving resources to address individual, interpersonal, and system-level factors that contribute to couples’ adjustment to ADRD diagnoses.

